# MiR-338-5p enhances the radiosensitivity of esophageal squamous cell carcinoma by inducing apoptosis through targeting survivin

**DOI:** 10.1038/s41598-017-10977-9

**Published:** 2017-09-07

**Authors:** Misun Park, Hyeon-joon Yoon, Moon Chul Kang, Junhye Kwon, Hae Won Lee

**Affiliations:** 1 Department of Translational Research, Korea Cancer Center Hospital, Korea Institute of Radiological and Medical Sciences, Seoul, Korea; 2 Department of Radiological & Medico-Oncological Sciences, Korea University of Science and Technology, Daejeon, Korea; 3 Department of Thoracic Surgery, Korea Cancer Center Hospital, Korea Institute of Radiological and Medical Sciences, Seoul, Korea

## Abstract

Radioresistance is a challenge in the treatment of esophageal squamous cell carcinoma (ESCC). MicroRNAs (miRNAs) are known to play an important role in the functional modification of cancer cells and recent studies have reported miRNA-mediated radiotherapy resistance. However, further research is necessary to reveal the regulation mechanisms, and treatment strategies using miRNA are yet to be established for ESCC. We compared the miRNA expression profiles of ESCC parental (TE-4) and acquired radioresistance (TE-4R) cell lines using a miRNA microarray and qRT-PCR. Our data showed that miR-338-5p, one of the target miRNA biomarkers, was significantly downregulated in TE-4R. Ectopic overexpression of miR-338-5p induced apoptosis and sensitivity to radiation treatment by interfering with survivin, which is a known inhibitor of apoptosis. Overexpression of survivin reversed miR-338-5p-induced apoptosis. Tumor xenograft experiments indicated that therapeutic delivery of the miR-338-5p mimics via direct injection into tumor mass increased sensitivity to radiation therapy. In conclusion, our findings suggest that miR-338-5p is a potential radiosensitizer and may be a therapeutic biomarker for radioresistant in ESCC.

## Introduction

Esophageal squamous cell carcinoma (ESCC) is one of the deadliest cancers, with high incidence in East Asian countries^1^. Surgical resection is the primary treatment of ESCC and provides the best chance for cure. Although the 5-year survival rate remains poor, chemo-radiotherapy is of utmost importance in some cases^2, 3^. One of the recommended treatments for ESCC is radiotherapy; however, therapeutic outcomes are not satisfactory because of tumor radioresistance owing to various mechanisms such as the DNA repair pathway and hypoxic cancer stem cells^4–6^. Accumulating evidence suggests that cell apoptosis may play important roles in regulating the response to radiotherapy; however, its specific contribution remains unknown^7–9^. Clarification of these molecular mechanisms can help identify important molecular targets that must be selectively controlled to improve the effectiveness of radiotherapy. Studies on the molecular mechanisms involved in the regulation of radioresistance revealed an association between apoptosis and tumor radiosensitivity, suggesting that apoptotic cell death may play an important role in determining the response to radiation in various cancers^10–14^. Although these studies have improved our understanding of the molecular mechanisms underlying radioresistance, the detailed mechanisms in radioresistant cell lines remain unknown and *in vivo* studies of radiation-induced apoptosis in ESCC have not been reported.

MicroRNAs (miRNAs) are small noncoding RNAs that regulate gene expression by binding to the 3′ untranslated region (UTR) of their target in a sequence-specific manner, thereby decreasing gene expression^15, 16^. Accumulating evidence indicates that miRNA expression is involved in tumorigenesis and cancer biology^17, 18^. Several miRNAs have been correlated with patient survival and may be useful in the prediction and modification of anticancer treatments, including radiotherapy^19–21^. However, miRNA expression profiling data of radioresistant ESCC cell lines are limited and potential miRNA functions in therapeutic strategies remain unclear.

In this study, we predicted that miR-338–5p is a regulator of radioresistance in ESCC based on miRNA expression profiling of ESCC parental (TE-4) and acquired radioresistance (TE-4R) cell lines. We checked for binding sites for miR-338-5p in the 3′ UTR of survivin, one of the key regulators of apoptosis inhibition. Based on our findings, we report that miR-338-5p increases radiation-induced apoptosis and enhances radiosensitivity by downregulating survivin expression.

## Results

### Establishment of a radioresistant ESCC cell line

**Figure 1:**
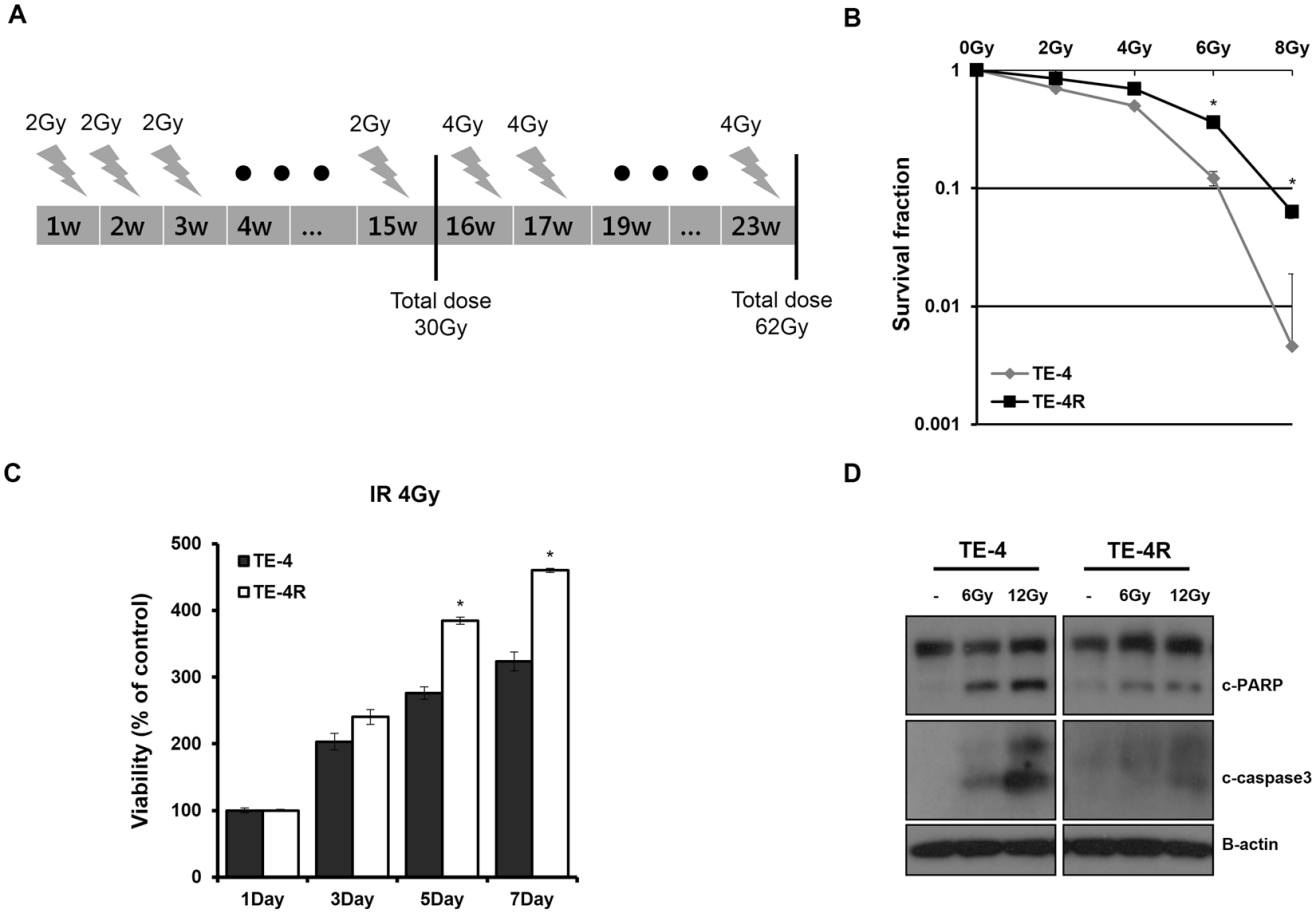
Establishment and validation of the ESCC radioresistance cell line. (**A**) Schematic representation of the generation of radioresistant cell line (TE-4R) from parental TE-4 cells. (**B**) TE-4 and TE-4R cells were irradiated with 2, 4, 6, or 8 Gy radiation doses. Colonies that formed after a 2-week incubation were stained with crystal violet and counted using ImageJ software. (**C**) Cell viability was analyzed by MTS at different time points after irradiation with 4 Gy. Data in the bar chart are the mean ± SD of three independent experiments (*p* < 0.05). (**D**) TE-4 and TE-4R cells were treated with the indicated doses of radiation and analyzed for the expression of cleaved PARP and caspase3 by western blotting. *G*y gray, *c-PARP* cleaved poly (ADP-ribose) polymerase, *c-caspase3* cleaved caspase3, *IR* ionizing radiation.

To establish a radioresistant ESCC cell line, we used γ-rays to select radioresistant populations from parental TE-4 ESCC cells. TE-4 cells were exposed to fractionated radiation and surviving cells formed colonies. The colonies were pooled, and the radiation treatment was repeated (Fig. 1A). Cells derived from this selection were named TE-4R. To determine the radiosensitivity of TE-4R cells as compared with parental TE-4 cells, a clonogenic survival assay was performed. The surviving fraction of TE-4R cells was significantly larger than that of the TE-4 cells at various doses (Fig. 1B). Cell viability analysis by MTS assay at different time points (1, 3, 5, and 7 days) after irradiation with 4 Gy showed that TE-4R viability was significantly higher at day 5, and even more markedly so at day 7 after irradiation (Fig. 1C) (p < 0.05). To evaluate the cellular response to radiation, the levels of apoptosis-related proteins were analyzed. Cleavaged Poly(ADP-ribose) polymerase (PARP) and caspase3, which is considered to be a hallmark of apoptosis, was elevated in TE-4 cells than TE-4R cells as indicated by increased levels of cleaved PARP and caspase3 in the former (Fig. 1D). These data indicated that TE-4R cells are more resistant to cell death than the parental cells after irradiation.

### MiR-338-5p is downregulated in radioresistant ESCC cells

**Figure 2:**
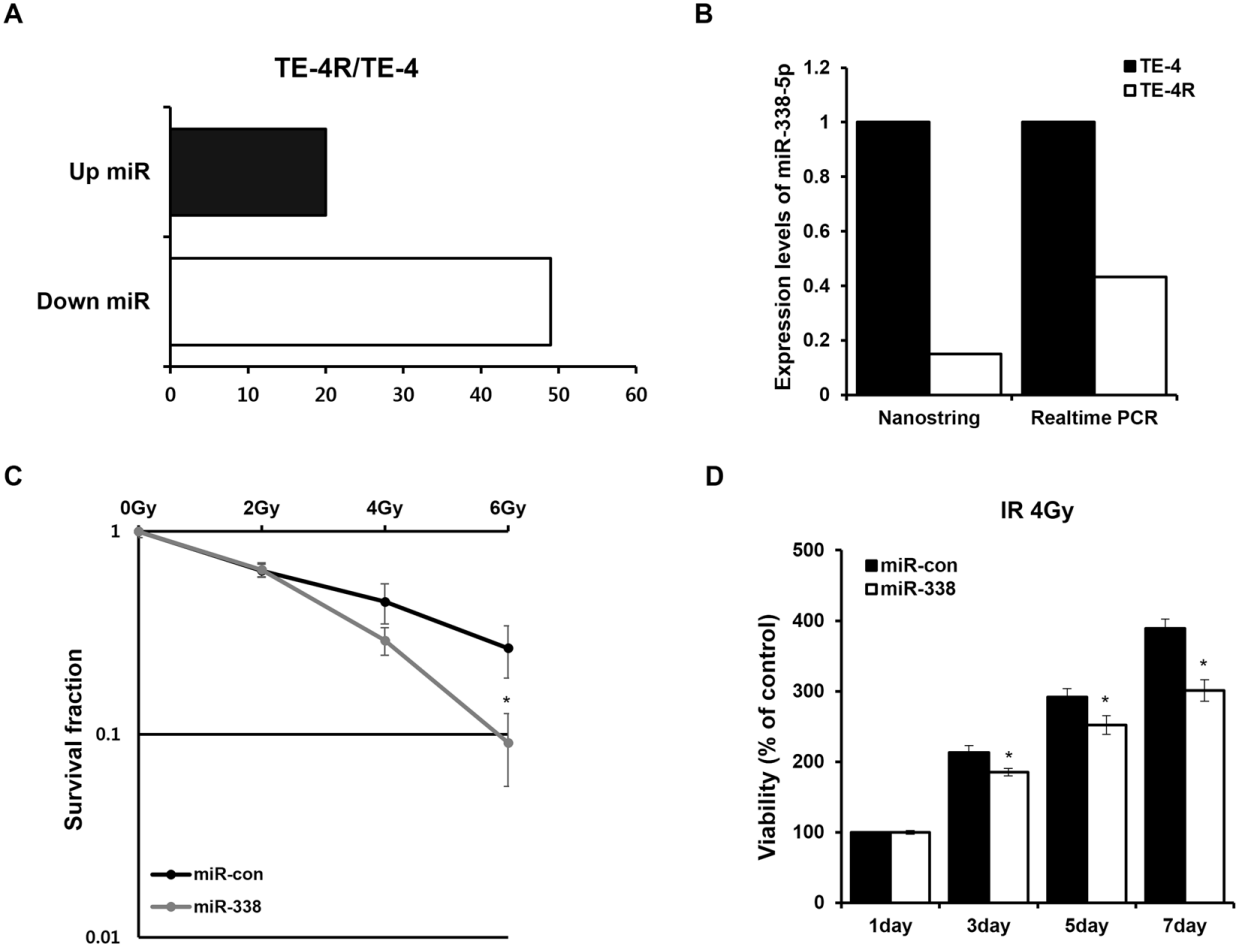
MiR-338-5p increases the sensitivity of TE-4 cells to radiation. (**A**) MiRNAs differentially expressed between TE-4 and TE-4R were analyzed using NanoString nCounter miRNA Expression Assays. (**B**) MiR-338-5p expression levels were measured by NanoString nCounter miRNA Expression Assays and real-time PCR in TE-4R. RNU6B was used as an internal control for real-time PCR. (**C**) TE-4 cells were transfected with control or miR-338-5p mimic and irradiated with 2, 4, or 6 Gy. Clonogenic survival assays performed and surviving fractions were fitted to the linear-quadratic equation. (**D**) TE-4 cells transiently transfected with control or miR-338-5p mimic were exposed to 4 Gy radiation and cell viability was analyzed using the MTS cell proliferation assay at 1, 3, 5, and 7 days after irradiation. Data in the bar chart are the mean ± SD of three independent experiments (*p* < 0.05).

To identify miRNAs that regulate radiosensitivity in ESCC cell lines, we used the NanoString nCounter miRNA expression assay. The miRNA expression array indicated that 20 miRNA were increased (fold change > 1.5) and 49 miRNA were decreased (fold change < 1.5) in TE-4R cells as compared to their parental cell line (Fig. 2A). We selected miR-338-5p for further analysis because its function and mechanism in radiosensitization have not been characterized. MiR-338-5p was downregulated in the TE-4R cell line (fold change = −5.1). Real-time PCR analysis confirmed the microarray data (Fig. 2B). To investigate the effect of miR-338-5p as a radiosensitizer, TE-4 cells were transiently transfected with control (miR-con) or miR-338-5p mimic (miR-338), and their survival after irradiation was determined. Western blot results confirmed that transfection was successful (Fig. S1). A clonogenic survival assay showed that miR-338-5p significantly decreased the surviving cell fraction as compared to miR-con (Fig. 2C). The effect of miR-338-5p expression on cell growth after irradiation was also examined by MTS assay; miR-338-5p-treated cells showed reduced numbers as compared to miR-con-treated cells at 3, 5, and 7 days (*p* < 0.05) (Fig. 2D). Taken together, these results demonstrated that miR-338-5p expression in ESCC cells significantly decreases radioresistance *in vitro*; thus, miR-338-5p can sensitize ESCCs cells to irradiation.

### MiR-338-5p inhibits radiation-induced apoptosis

**Figure 3:**
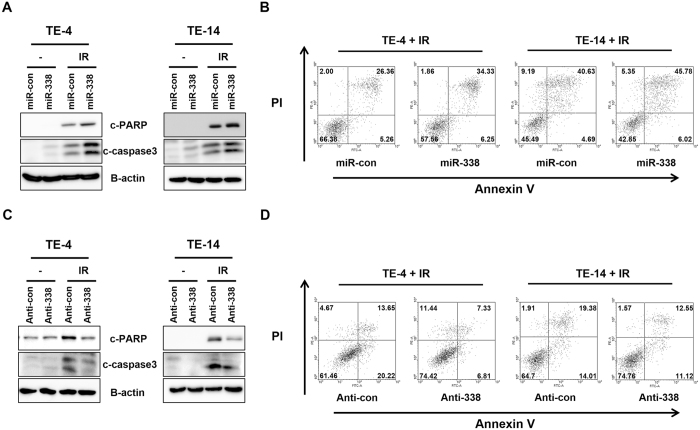
MiR-338-5p promotes radiation-induced apoptosis in ESCC cells. TE-4 cells were transfected with miR-338, anti-338, or control miRNA prior to subsequent experiments. (**A,C**) Cells were harvested at 48 h after irradiation (0 or 6 Gy) and analyzed by western blotting using antibodies against c-PARP and c-caspase3. β-actin served as a loading control. (**B,D**) Apoptosis was assayed by flow cytometry at 48 h after irradiation with 6 Gy.

Our and other groups have shown that apoptosis is one of the major factors regulating radioresistance in ESCC^9, 12, 13^. To investigate whether miR-338-5p acts as a radiosensitizer through regulation of apoptosis, we examined apoptosis following irradiation. MiR-338-5p-transfected TE-4 and TE-14 cells showed significant increases in cleaved PARP and caspase3 as compared to controls after irradiation, from 31.62% to 40.58% in TE-4 and from 45.32% to 51.8% in TE-14 (Fig. 3A,B). To confirm the apoptotic effect of miR-338-5p, we inhibited miR-338-5p using miR-338 inhibitor (Anti-338). Anti-338 repressed cleaved PARP and caspase3 expression after irradiation in both cell lines (Fig. 3C). Furthermore, anti-338-transfected groups decreased from 33.87% to 15.94% for TE-4 and from 33.39% to 23.67% for TE-14 (Fig. 3D). On the basis of these findings, we concluded that miR-338-5p sensitizes ESCC cells through enhanced apoptosis.

### MiR-338-5p regulates the expression of survivin

**Figure 4:**
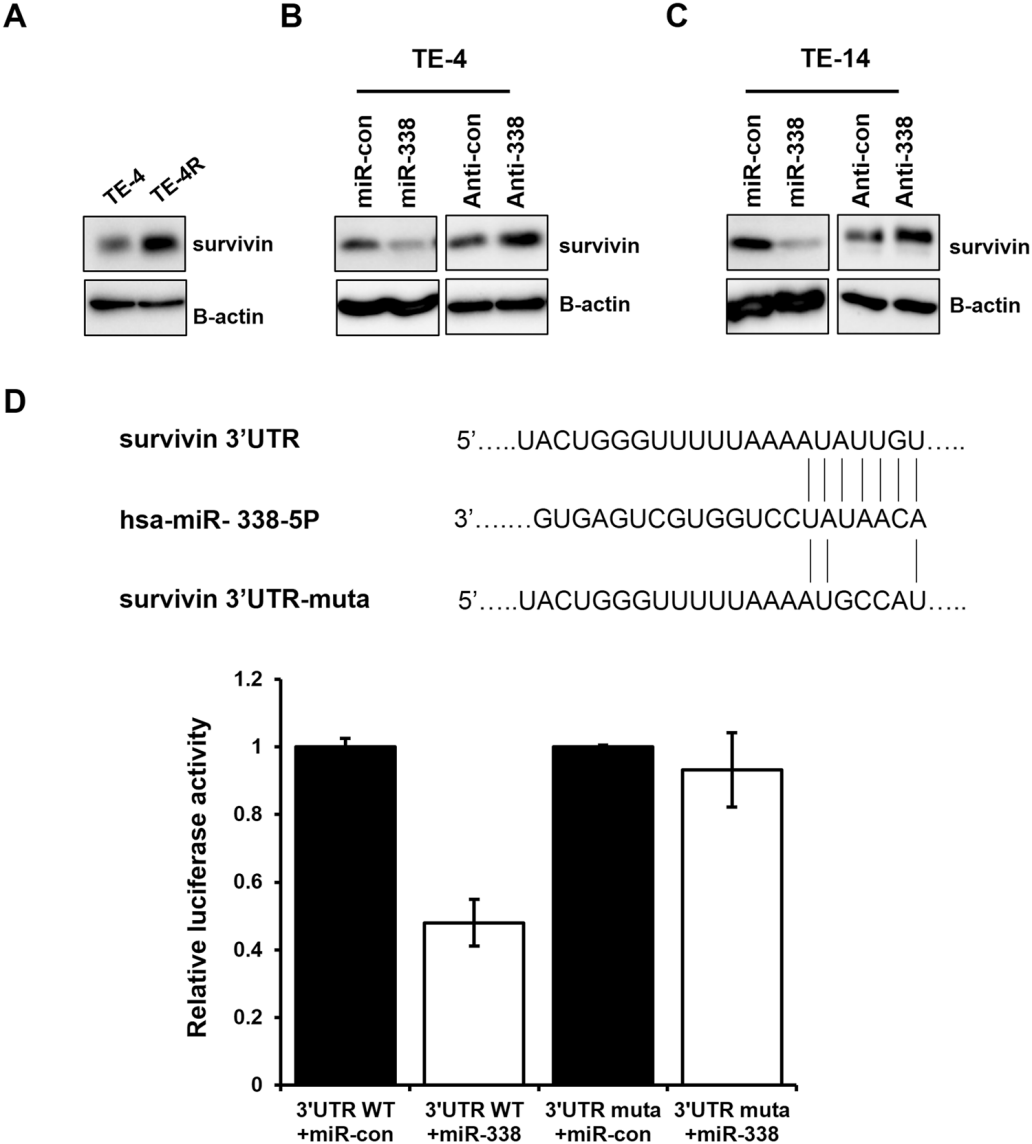
Survivin is a direct target of miR-338-5p in ESCC cells. (**A**) Levels of survivin were compared in parental TE-4 cells and radioresistance TE-4R cells. β-actin served as a loading control. (**B,C**) MiR-338, anti-338, or control miR were transfected into TE-4 and TE-14 cells. After 72 h, expression levels of survivin and β-actin were analyzed by western blot analysis. (**D**) Top, the predicted miR-338-5p-binding sites in the survivin (3′ UTR WT) and mutant (3′ UTR muta) 3′ UTR are shown; down, 3′ UTR WT or 3′ UTR muta dual luciferase reporter vector was cotransfected into TE-4 cells with miR-con or miR-338, and firefly luciferase activity normalized to Renilla luciferase activity is shown. Data are the means ± SD, *p* < 0.05.

To understand how miR-338-5p modulates radiation-induced apoptosis, we analyzed target genes of miR-338-5p predicted by TargetScan (www.targetscan.org) and miRanda (www.microrna.org). Most notably, one of the predicted targets is survivin, which is known to inhibit apoptosis. Previous studies have suggested that survivin is important in the development of radioresistance through the regulation of apoptosis^22–25^. When compared with TE-4 cells, TE-4R cells exhibited much higher survivin expression (Fig. 4A). Thus, we assumed that miR-338-5p targets survivin. To analyze the targeted regulation of survivin by miR-338-5p in ESCC cells further, the miR-338-5p level was manipulated by transfecting the cells with miR-338-5p mimic and inhibitor. MiR-338-5p mimic transfection significantly reduced survivin expression, whereas its expression increased in anti-338-transfected cells as compared with their controls, in both the TE-4 and TE-14 cell lines (Fig. 4B,C). To confirm survivin as a direct target of miR-338-5p, we constructed a luciferase reporter plasmid containing the binding site of the survivin 3′ UTR (3′ UTR WT) and one harboring a deletion of the binding site (3′ UTR muta). We co-transfected these plasmids with miR-con or miR-338 into TE-4 cells. The results revealed significantly reduced luciferase activity in cells transfected with 3′ UTR WT- and miR-338, whereas the mutant form did not affect luciferase activity (Fig. 4D). These findings suggested that survivin is a direct target of miR-338-5p.

### Survivin overexpression rescues cells from apoptosis induced by miR-338-5p

**Figure 5:**
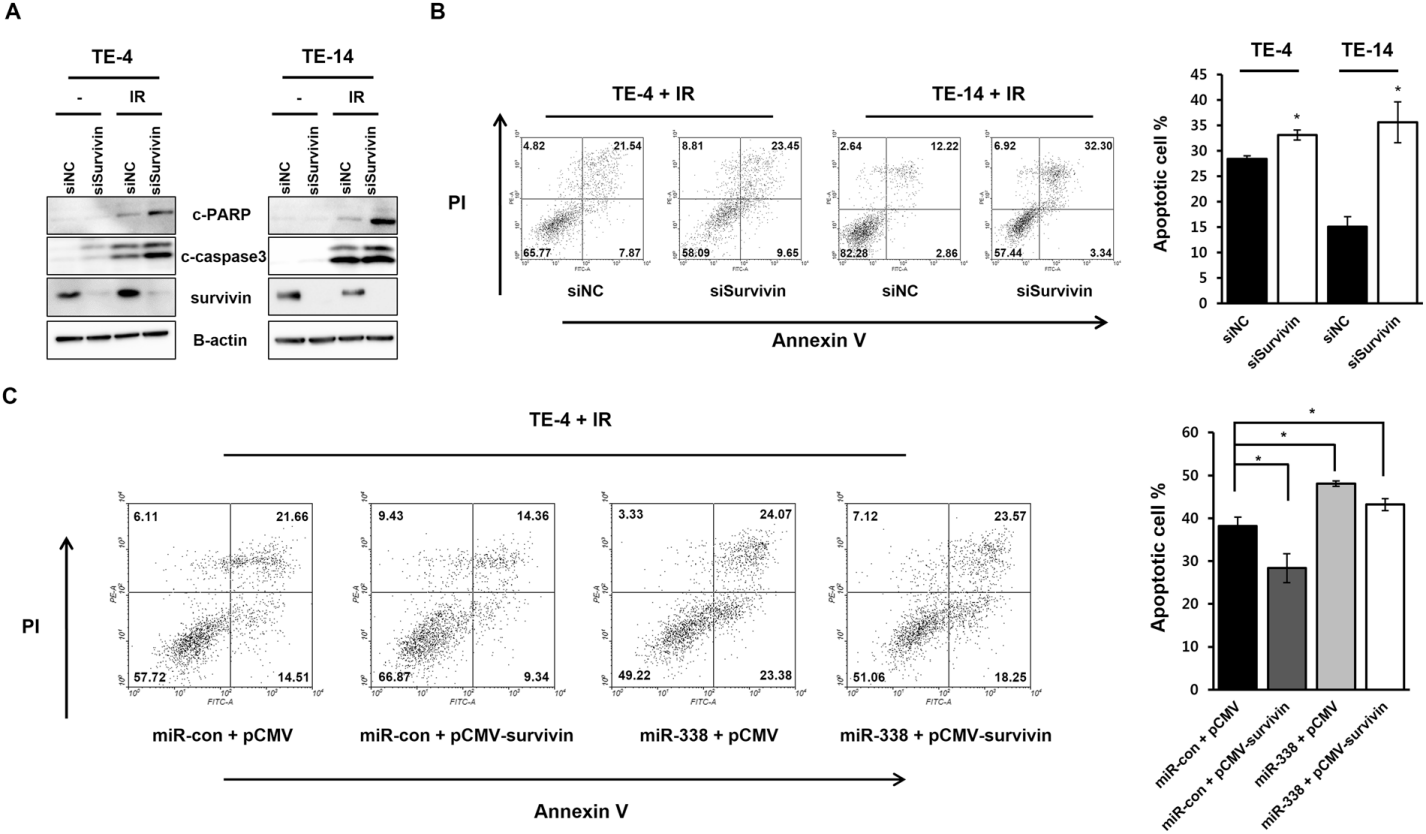
Survivin overexpression rescues cells from apoptosis induced by miR-338-5p. TE-4 and TE-14 cells were transfected with survivin siRNA (siSurvivin) or control siRNA (siNC) prior to subsequent experiments. (**A**) Cells were harvested at 48 h after irradiation (0 or 6 Gy) and analyzed by western blotting using c-PARP, survivin, and c-caspase3 antibodies. β-actin served as a loading control. (**B**) Apoptosis was assayed by flow cytometry at 48 h after irradiation with 6 Gy. The graph indicates the apoptotic ratios of annexin-V-FITC- and PI-positive cells. (**C**) Left, TE-4 cells were transfected with the miR-338 mimic in the presence of survivin or control vector. Next day, the cells were irradiated with 6 Gy and apoptosis was assayed using flow cytometry after 48 h. Right, the graph indicates the apoptotic ratios of annexin-V-FITC- and PI-positive cells. Data in the bar chart are the mean ± SD of three independent experiments (*p* < 0.05).

To confirm the interaction between miR-338-5p and survivin, we silenced the latter using siRNA and examined the effect on apoptosis. Western blot assay and flow cytometry analysis of Annexin V-stained cells showed that the knockdown of endogenous survivin expression could imitate the effects associated with miR-338-5p overexpression, including increased cleaved PARP and caspase3 expression levels and promotion of apoptosis (Fig. 5A and B). To clarify whether apoptosis induction by miR-338-5p is mediated through suppressing survivin expression, a pCMV survivin expression plasmid was constructed. A rescue experiment was conducted to explore whether survivin could functionally reverse radiation-induced apoptosis by miR-338-5p. The pCMV-survivin vector and pCMV control vector were transfected into TE-4 cells with miR-con or miR-338-5p, and the cells were examined by flow cytometry after irradiation. The level of apoptotic cells was decreased in cells overexpressing survivin as compared to control cells transfected with miR-338-5p (Fig. 5C). This rescue experiment revealed that survivin can reverse the apoptosis induced by miR-338-5p.

### MiR-338-5p enhances radiosensitivity of ESCC in xenograft models

**Figure 6:**
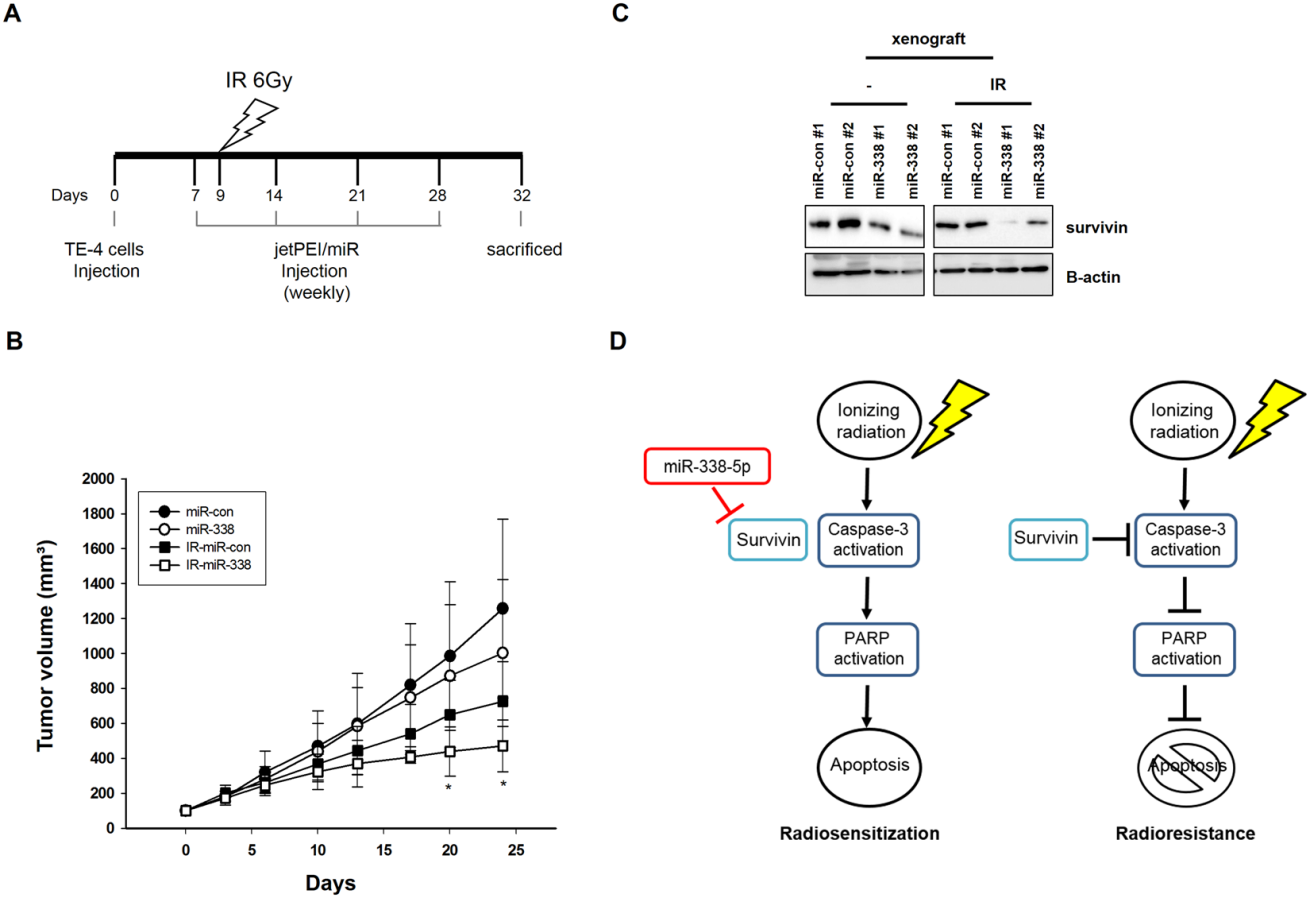
Upregulation of miR-338-5p decreases TE-4 cell radioresistance *in vivo*. (**A**) Schedule of administration of miR-338-5p to and irradiation of TE-4 xenografts. TE-4 cells were inoculated into the backs of nude mice (n = 9), and when tumor volumes reached 100 mm^3^, treatment by intratumoral injection of 10 μg jetPEI/miR-con or jetPEI/miR-338 complexes was started. Arrows show the days on which injections of jetPEI/miR complex were administered. (**B**) Tumor volumes expressed as percentages (%) relative to the volume at the time of the first miR injection (day 0). Error bars indicate the SD (n = 9). (**C**) On day 26 after injection, the mice were sacrificed and survivin expression was detected by western blotting. (**D**) Schematic illustration of the difference between radiosensitivity and radioresistance in ESCC.

To explore whether miR-338-5p enhances radiosensitivity *in vivo*, we generated subcutaneous tumors in nude mice using TE-4 cells. As illustrated in Fig. 6A, control or miR-338-5p mimic were injected into the tumors 4 times at 1-week intervals before and after ionizing radiation of 6 Gy, and tumor size was measured every 3 days. After irradiation, control tumors continued to increase in volume; however, miR-338-5p-injected TE-4 tumors grew evidently slower (Fig. 6B). Additionally, survivin expression was downregulated in miR-338-transfected xenografts (Fig. 6C). Taken together, these results demonstrated that miR-338-5p expression obviously decreases radioresistance *in vivo*, suggesting that *in vivo* administration of miR-338-5p mimic has considerable potential for radiosensitization.

## Discussion

Radiotherapy plays an important role in the treatment of esophageal cancers as part of combined-modality therapy together with chemotherapy and surgery. This treatment approach has improved tumor control and survival rates. However, tumor recurrence because of radioresistance occurs in a high proportion of patients; therefore, therapeutic strategies to improve the response to radiation in ESCC need to be developed. Here, we identified miR-338-5p as a radiosensitizer. Additionally, we found the miR-338-5p target survivin to be an important inhibitor of radiation-induced apoptosis (Fig. 6D).

Abundant miRNAs regulate a wide range of biological processes in cancers. Using a miRNA expression profiling approach, we identified miR-338-5p as a downregulated miRNA in radioresistant ESCC. In a previous study, miR-338-5p was considered to function as an oncomiR by inhibiting PIK3C3 expression and autophagy in colorectal cancer^26^. However, miR-338-5p might have different functions in different cancers. Our results demonstrated that miR-338-5p acts as a radiosensitizer through inhibition of apoptosis in ESCC. Results from several other groups have confirmed miRNAs to be involved in the radioresistance of ESCC. Huang *et al*. identified miR-21 as an activator of radioresistance through inhibition of PTEN^27^, and Su *et al*. recently found a set of miRNAs aberrantly expressed in acquired radioresistant ESCC cells that conferred resistance through the Wnt/B-catenin signal pathway^21^. Although these studies reported on miRNAs associated with radioresistance in ESCC, our study is the first to show that miR-338-5p as radiosensitizer inhibits radiation-induced apoptosis *in vitro* and *in vivo*.

We showed that survivin expression was markedly enhanced in the radioresistant cell line as compared to the parental cells. We and others previously showed that survivin is associated with radioresistance in pancreatic cancer, rectal cancer, head and neck squamous carcinoma, as well as lung cancer^22, 23, 28, 29^. Survivin is tightly associated with stemness-promoting pathways such as Notch^30^, Oct4, and STAT3^31^. Moreover, survivin was shown to be a major target of different pro-survival signaling pathways, such as the PI3K/AKT pathway^32^. These pathways are known to control radiosensitivity; thus, survivin might induce radioresistance in ESCC through the regulation of these signaling pathways. Additionally, accumulation of survivin and interaction with components of the DNA-double-strand break (DSB) repair machinery in glioblastoma cells indicated that survivin regulates DSB repair, significantly improving survival in these cells^25^. It will be interesting to study the different roles of survivin, including not only the inhibitory effect on caspase activity during apoptosis but also functions in DNA damage repair in ESCC radioresistant cells. Future studies are required to test this hypothesis.

To the best of our knowledge, this is the first report of the roles of miR-338-5p and survivin in radioresistance of ESCC. We reported a novel role for miR-338-5p as a key regulator of apoptosis and showed that miR-338-5p sensitizes ESCC to irradiation by inhibiting survivin expression suggesting miR-338-5p as a potential therapeutic strategies for improving the response of ESCC patients to radiation therapy.

## Methods

### Establishment of a radioresistant cell line and cell culture

TE-4 and TE-14 ESCC cells were purchased from RIKEN (RIKEN, Saitama, Japan). To establish a radioresistant TE-4 cell line, cells were treated with 2 Gy per week for 15 weeks. The surviving cells were treated with 4 Gy per week for 15 more weeks. Upon completion of the radiation treatment, surviving cells were defined as the radioresistant TE-4 cell line, named TE-4R. All ESCC cells were cultured in RPMI-1640 (Corning, New York, NY, USA) supplemented with 10% FBS (Gibco, Grand Island, NY, USA) and antibiotics (100 units/ml penicillin and 100 μg/ml streptomycin, Corning) at 37 °C in an atmosphere of 5% CO_2_/95% air.

### Irradiation and clonogenic assay

TE-4 and TE-4R cells were seeded at 2 × 10^3^ cells per well in 6-well culture plates, cultured for 24 h, and irradiated with different radiation doses (0, 2, 4, 6, and 8 Gy) using a ^137^Cs γ-ray source (Atomic Energy of Canada Ltd, Chalk River, Canada) at a dose rate of 3.81 Gy/min. After 14 days of culture, the cells were fixed, stained with 2% crystal violet, and cell colonies were counted using the ImageJ software.

### Transfection

One day before transfection, cells were seeded in 6-well plates and cultured to 60–80% confluence. Then, the cells were treated with 50 nM control microRNA, miR-338-5p mimic; 20 nM siNC or siSurvivin (Bioneer, Daejeon, Korea); or 1 μg survivin expression plasmid or vehicle (Origene, Rockville, MD, USA) using Lipofectamine 2000 (Invitrogen, Carlsbad, CA, USA). After 6 h, the cells were transferred to complete growth medium. On the third day after transfection, the cells were harvested and used in further experiments.

### MiRNA expression profiling using a NanoString array

MiRNAs were prepared using a miRNeasy Mini kit (Qiagen, Hilden, Germany) and hybridized to the array in the nCounter miRNA Expression Assay (NanoString Technologies, Seattle, WA, USA) following the manufacturer’s instructions.

### Real-time PCR

MiRNAs were quantified using the miScript SYBR Green PCR kit (Qiagen) with specific primers for miR-338-5p and U6 (Qiagen). Quantitative PCRs were run on a 7500 Real-Time PCR system (Applied Biosystems, Foster City, CA, USA). The reaction mixtures were incubated at 95 °C for 10 min, followed by 40 cycles of 95 °C for 15 s, 60 °C 30 s, and 72 °C 30 s. The expression level was determined by the 2^−∆∆Ct^ method.

### Flow cytometry analysis of apoptosis

Irradiated cells were stained with fluorescein isothiocyanate (FITC)-labeled annexin V/propidium iodide (PI) using an apoptosis detection kit (BD Biosciences, San Jose, CA, USA) according to the manufacturer’s instructions, and were analyzed by flow cytometry (BD Biosciences).

### Western blotting

For western blot analysis, cells were washed with cold PBS and lysed in RIPA buffer (Thermo Fisher Scientific, Grand Island, NY, USA). Proteins were quantified using the Bradford method and equal amounts of protein were resolved by SDS-PAGE and analyzed by western blotting as previously described^33^. The membranes were incubated with primary antibodies against cleaved PARP, caspase3, survivin (Cell Signaling Technology, Beverly, MA, USA) and β-actin (Sigma-Aldrich, St. Louis, MO, USA) overnight at 4 °C and with a secondary antibody (Santa Cruz Biotechnology, Santa Cruz, CA, USA) for 1 h at room temperature. Proteins were visualized using enhanced chemiluminescence (Thermo Fisher Scientific). Western blot images were analyzed with a LAS-4000 mini (GE Healthcare Life Sciences, Uppsala, Sweden) and Bio-Rad ChemiDoc (Bio-Rad, Richmond, CA, USA).

### Cell proliferation assay

Cells were seeded in 96-well plates and allowed to attach for 24 h. Then, the cells were irradiated with 4 Gy. At various time points, 10 μl of (3-(4,5-dimethylthiazol-2-yl)-5-(3-carboxymethoxyphenyl)-2-(4-sulfophenyl)-2H-tetrazolium; MTS) of the CellTiter 96 AQueous One Solution Cell Proliferation Assay kit (Promega, Madison, WI, USA) was added to 100 μl of culture media for 1 h. The absorbance at 490 nm was read using a plate reader.

### Dual luciferase reporter assay

TE-4 cells were seeded into 6-well plates one day before transfection. The cells were transfected with 1 μg of pmiRGLO vector (Promega) containing predicted miR-338-5p-binding sites of the survivin 3′ UTR and 50 nM microRNA mimic using Lipofectamine 2000. pRL-TK, which encodes Renilla luciferase, was included in all transfections to normalize for transfection efficiency. At 48 h after transfection, assays were performed using a dual luciferase reporter system (Promega) according to manufacturer’s instructions. Firefly luciferase activity was normalized to that of Renilla, and then compared with those of the respective controls.

### Animal experiments

Female nude mice at 6 weeks of age were subcutaneously injected with 1 × 10^7^ TE-4 cells into the right flank of the back. At 7 days post-injection, 1.6 μl JetPEI and 10 µg miR-con or miR-338-5p were directly injected into the tumor, and injections were repeated every week for 4 weeks. Then, the mice were irradiated with a single dose of 6 Gy using an X-RAD 320 X-ray irradiator (Softex, Goyang, Korea) at a dose rate of 2 Gy/min. This study was carried out in accordance with relevant guidelines and regulations. The animal experiments were reviewed and approved by the Institutional Animal Care and Use Committee of Korea Institute of Radiological and Medical Sciences (protocol number: KIRAMS 2016-0032). The animal care facility is accredited by the Association for Assessment and Accreditation of Laboratory Animal Care.

### Statistical analysis

Data obtained from at three experiments are expressed as the mean ± standard deviation. Statistical significance of differences was analyzed by Student’s *t*-test, and *p* < 0.05 was considered significant.

## Electronic supplementary material

supplementary figure
