# Supplementary material for: MiR-338-5p enhances the radiosensitivity of esophageal squamous cell carcinoma by inducing apoptosis through targeting survivin
Source: Sci Rep. 2017 Sep 7;7:10932. doi: 10.1038/s41598-017-10977-9 (PMC5589838; doi:10.1038/s41598-017-10977-9)
Supplement: Supplementary file 1 — supplementary figure [file 41598_2017_10977_MOESM1_ESM.pdf]

MiR-338-5p enhances the radiosensitivity of esophageal squamous cell carcinoma by inducing apoptosis through targeting survivin

Misun Park, Hyeon-joon Yoon, Moon Chul Kang, Junhye Kwon, Hae Won Lee

supplementary figure 1

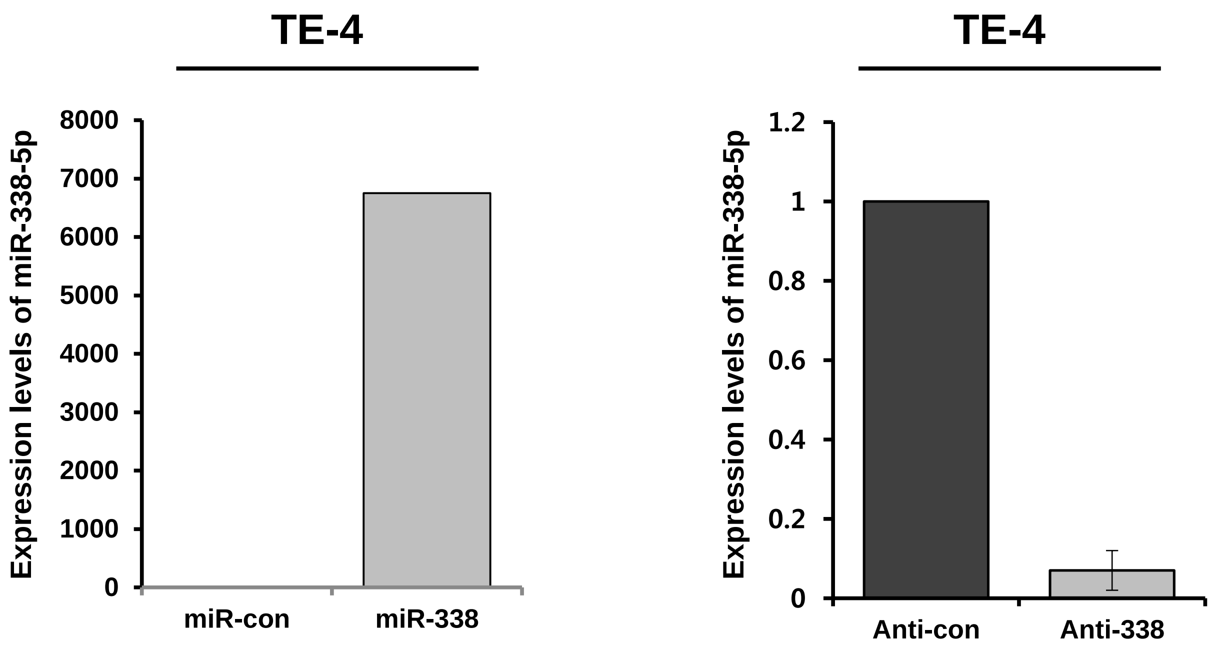

Supplementary Fig. 1. Expression of miR-338-5p was determined by real-time PCR in TE-4 cells 48h after transfection with miR-338-5p mimic (miR-338) and inhibitor (Anti-338). RNU6B was used as an internal control for real-time PCR. Data in the bar chart are the mean ± SD of three independent experiments.

MiR-338-5p enhances the radiosensitivity of esophageal squamous cell carcinoma by inducing apoptosis through targeting survivin

Misun Park, Hyeon-joon Yoon, Moon Chul Kang, Junhye Kwon, Hae Won Lee

supplementary figure 2

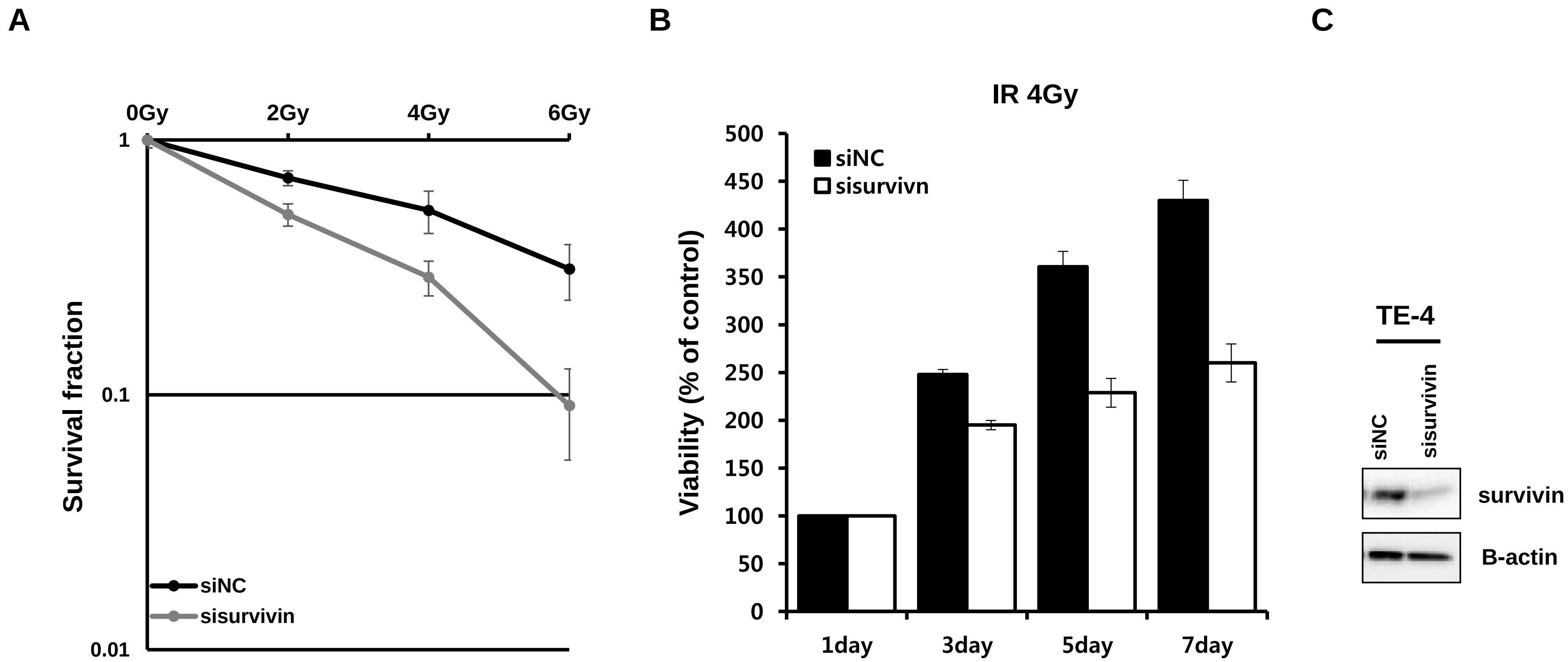

Supplementary Fig. 2. Inhibition of survivin increases the sensitivity of TE-4 cells to radiation. (**A**) TE-4 cells were transfected with control or sisurvivin and irradiated with 2, 4, or 6 Gy. Clonogenic survival assays performed and surviving fractions were fitted to the linear-quadratic equation. (**B**) TE-4 cells transiently transfected with control or sisurvivin were exposed to 4 Gy radiation and cell viability was analyzed using the MTS cell proliferation assay at 1, 3, 5, and 7 days after irradiation. Data in the bar chart are the mean ± SD of three independent experiments ($p < 0.05$). (**C**) sisurvivin and control was transfected into TE-4. After 72 h, expression levels of survivin and β-actin were analyzed by western blot analysis.
